# Isolation of Volatile Compounds with Repellent Properties against *Aedes albopictus* (Diptera: Culicidae) Using CPC Technology

**DOI:** 10.3390/molecules26113072

**Published:** 2021-05-21

**Authors:** Anastasia Liakakou, Apostolis Angelis, Dimitrios P. Papachristos, Nikolas Fokialakis, Antonios Michaelakis, Leandros A. Skaltsounis

**Affiliations:** 1 Laboratory of Pharmacognosy and Natural Products Chemistry, School of Pharmacy, University of Athens, Panepistimioupoli, Zografou, 15771 Athens, Greece; aliakakou@pharm.uoa.gr (A.L.); aangjel@pharm.uoa.gr (A.A.); fokialakis@pharm.uoa.gr (N.F.); 2 Scientific Directorate of Entomology and Agricultural Zoology, Benaki Phytopathological Institute, 14561 Kifissia, Greece; d.papachristos@bpi.gr (D.P.P.); a.michaelakis@bpi.gr (A.M.)

**Keywords:** repellent activity of essential oils, centrifugal partition chromatography, *Pinus* and *Juniperus* spieces, Asian tiger mosquito, limonene, guaiol and germacrene

## Abstract

The present work describes the use of Centrifugal Partition Chromatography (CPC) for the bio-guided isolation of repellent active volatile compounds from essential oils. Five essential oils (EOs) obtained from three *Pinus* and two *Juniperus* species were initially analyzed by gas chromatography–mass spectrometry (GC/MS) and evaluated for their repellent properties against *Aedes albopictus.* The essential oil from needles of *P. pinea* (PPI) presented the higher activity, showing 82.4% repellency at a dose of 0.2 μL/cm^2^. The above EO, together with the EO from the fruits of *J. oxycedrus* subsp. *deltoides* (JOX), were further analyzed by CPC using the biphasic system n-Heptane/ACN/BuOH in ratio 1.6/1.6/0.2 (*v/v/v*). The analysis of PPI essential oil resulted in the recovery of (−)-limonene, guaiol and simple mixtures of (−)-limonene/*β*-pheladrene, while the fractionation of JOX EO led to the recovery of *β*-myrcene, germacrene-D, and mixtures of *α*-pinene/*β*-pinene (ratio 70/30) and *α*-pinene/germacrene D (ratio 65/45). All isolated compounds and recovered mixtures were tested for their repellent activity. From them, (−)-limonene, guaiol, germacrene-D as well the mixtures of (−)-limonene/*β*-pheladrene presented significant repellent activity (>97% repellency) against *Ae. albopictus*. The present methodology could be a valuable tool in the effort to develop potent mosquito repellents which are environmentally friendly.

## 1. Introduction

Mosquitoes are responsible for spreading viruses, causing serious diseases such as malaria, filariasis, Japanese encephalitis, dengue fever and yellow fever. They cause the mortality of two million people every year and at least one million children die of diseases caused by mosquitoes [1]. *Aedes albopictus*, an invasive mosquito species, was spread in many countries worldwide, mainly due to the trade of used tires [2]. This mosquito species, also known as the Asian tiger mosquito, is responsible for the transmission of many human pathogens such as dengue, chikungunya, the Zika virus and filarial nematodes (*Dilofilaria* spp.). The protection from mosquito bites still remains the most effective way to prevent infections associated with those pathogens. The control of *Ae. albopictus* is very difficult and requires a concrete management plan which includes coordinated actions [3]. The lack of understanding of the proper integrated management of those arthropods makes the implemented measures ineffective. Consequently, personal protective measures against mosquito bites, such as the use of repellents, is necessary to protect from nuisance and/or to prevent the transmission of mosquito-borne diseases.

N,N-diethyl-3-methylbenzamide (DEET) has been considered one of the most effective repellents [4]. While there have been reports due to toxic effects on children and pregnant women, the harmful effects are minimized when label instructions are properly followed [5,6,7]. Research interest to find new natural anti-mosquito agents has grown in the last decades [8]. Finding new plant-derived products against mosquitoes could help us to improve our arsenal against mosquitoes, and furthermore, to reverse the negative impacts on human health [9,10]. Several plant ingredients have been considered for evaluation as potential deterrents, repellents and insecticidal agents against several insect pest species [11,12]. Essential oils derived from plants and their major component terpenes, can be environmentally-friendly, have a low-risk profile for mammals and humans and might provide natural alternatives instead of using conventional insecticides [13]. Particularly for repellents, there is an effort to develop products less harmful for humans and the environment. Therefore, the research on natural products with strong bioactivity, such as essential oils, has been prioritized [14]. Previous studies employing native plants in Greece regarding the essential oils from *Juniperus* [15] and Greek *Pinus* [16] species have been investigated, showing repellent actions against the mosquito species *Ae. albopictus*. There are also few attempts that have evaluated individual volatile components using chemicals (obtained as standards), but not fractionated components [17].

The counter-current chromatography (CCC) is a liquid–liquid separation technique with numerous advantages compared to other chromatographic techniques, especially for the treatment of a large number of mixtures in an environmentally-friendly way [18]. It is based on the different distribution of substances in the two miscible phases and is used for the fractionation of complex mixtures of compounds with a wide range of polarities [19,20]. It is important to note that CCC is the most effective technique for the separation of high added-value compounds from unpolar extracts such as the phenolic fractions obtained from edible oils, triterpenic fractions of different gums, etc. [21,22]. Nevertheless, the search of the literature for the analysis of essential oils by counter-current chromatography revealed the presence of a very limited number of similar scientific works. Most of them are related to the fractionation of essential oils [23] or in the separation of specific volatile compounds [24,25,26,27] by using High-Speed Countercurrent Chromatography (hydrodynamic CCC). Only two scientific works were found focusing on the separation of essential oils using a Centrifugal Partition Chromatography (CPC) device (hydrostatic CCC). In the first one, Dang and et al. described the isolation and purification of six volatile compounds from essential oil (EO) of *Curcuma wenyujin* [28], while the second work referred to the separation of Patchouli alcohol from Patchouli oil [29]. In both cases, the analysis was performed on a preparative scale. It is important to note that, compared to HSCCC, the CPC technology can be also used for large-scale separations, thus offeringthe advantage of pilot and industrial-scale applications.

In the present study, a separation method based on Centrifugal Partition Chromatography (CPC) was developed, aiming at the bio-guided isolation of volatile components with repellent activity against *Ae. albopictus*. Initially, five essential oils (EOs) obtained from *Pinus* and *Juniperus* species have been analyzed in terms of their chemical content and evaluated as repellant agents against the Asian tiger mosquito (*Ae. albopictus*). Taking into account the chemical composition and repellent activity of the EOs, the next step of the study was focused on the isolation of the main volatile compounds from the needles of *P. pinea* and fruits *J. oxycedrus* subsp. *deltoides*. The analyses were performed using the elution–extrusion CPC method with a non-aqueous biphasic system and resulted in effective recovery of the target compounds. All isolated compounds were tested for their repellent activity and important results have emerged.

## 2. Results and Discussion

### 2.1. Chemical Composition and Repellent Activity of Essential Oils

#### 2.1.1. Isolation and GC Analysis of the Essential Oils

In the present work, five essential oils obtained from the wood of *P. nigra* subsp. *nigra* (PNI) and *P. heldreichii* (PHE), needles of *P. pinea* (PPI), and fruits of *J.* *turbinata* (JTU) and *J. oxycedrus* subsp. *deltoides* (JOX) were studied. The isolation of the essential oils was carried out using Microwave-Assisted Hydrodistillation (MAHD), a ‘‘green’’ technique that is used both at the research and industrial level due to efficient heating, fast energy transfer, and environmental friendliness [30]. The volume and yield of the recovered EOs are presented in Table S1 (supplementary materials).

All obtained oils were analyzed by GC-MS (Figure S1a–e: Supplementary Material). The major constituents of each EO are presented in Table 1, while the detailed chemical compositions are given in Table S2 (supplementary material). The GC-MS analysis resulted in the identification of 92.11%, 98.40%, 97.88%, 98.19% and 98.68% of constituents for PNI, PHE, PPI, JTU and JOX EOs respectively. The PNI essential oil was characterized by a wide range of volatile compounds. Totally 43 secondary metabolites were identified from which *α*-pinene (32.82%) and sandaracopimarinal (18.10%) were presented as major components, followed by *δ*-cadinene (5.23%) and *γ*-muurolene (4.93%) (Table 1). Moreover, 13 volatile compounds were presented in concentrations between 1–4% while the other constituents are presented in traces (<1%) (Table S1: Supplementary Material). The chemical composition of the above EO significantly differs from the previously published data for the EO of wood of *P. nigra* where limonene, citronellol, *β*-pinene and *α*-pinene were found to be the major constituents [31]. PHE essential oil is less complicated than the previous one, containing only 13 volatile compounds. From them, cis-*β*-terpineol (47.41%) constitutes the major compound, followed by longifolene (11.88%) thunbergol (9.61%) and *α*-terpineol (9.19%) (Table 1). The results of the above analysis differ considerably from the previously-published results for the essential oils from wood [32] and needles [33] of *P. heldreichii*. On the other hand, the analysis of PPI essential oil showed the presence of 18 volatile compounds from which limonene (45.45%) was presented as the major constituent followed by *β*-phelladrene (12.79%) and *α*-pinene (9.50%) (Table 1). These results are in accordance with the previously published data in witch limonene (39.05%), *β*-phelladrene (13.8%) and *α*-pinene (5.13%) were presented in similar concentrations [34].

The essential oil obtained from fruits of *J. turbinata* were characterized by a high abundance of α-pinene (81.72%). It is important to note that the species *J. turbinata* of E. Mediterranean and Greek origin was previously recorded as *J. phoenicea* [35]. Thus, the previous works regarding the GC-MS analysis of EOs from the fruits of *J. phoenicea*, published by Evergetis et al. [36] and Vourlioti-Arapi et al. [15], refer to the same plant material (fruits of *J.* *turbinata*) and showed that α-pinene is also the major volatile compound. On the other hand, *β*-myrcene (43.53%) is the main compound of EO from fruits of *J. oxycedrus* subsp. *deltoides* followed by germacrene D (23.77%) and *α*-pinene (15.65%). These results are in accordance with the previously-published data, where *β*-myrcene, germacrene D and *α*-pinene ware presented in 53.85%, 17.03% and 19.70%, respectively [15].

#### 2.1.2. Repellent Activity of the Essential Oils

All EOs were tested for their repellent activity against *Ae. albopictus* by numbering the mosquito landings on the uncovered area of the glove for 5 min [17]. The results of the bioassays are present in Table 2. PPI showed important repellent activity, presenting approximately 82% repellency at a dose of 0.2 μL/cm^2^. On the other hand, the JTU, PNI, PHE and JOX essential oils presented moderate or low repellent activity against the Asian tiger mosquito.

To the best of our knowledge, this is the first time that the repellent activity of the essential oils from the wood part of *P. nigra* subsp. *nigra* and *P. hedreichii* and the fruit of *J. oxycedrus* subsp. *deltoides* are tested against *Ae. albopictus*. On the other hand, the essential oil of the fruits of *J.* *turbinata* grown in different places in Greece has been evaluated before [15,37]. In all references, although the aerial parts of *J.* *turbinata* have a high amount of *α*-pinene, the rest of the constituents may differentiate and the results of the repellant are in accordance with our study. In contrast, Traboulsi and co-workers tested the EO from needles of *P. pinea* against the *Culex pipiens* mosquito species and found that this EO was effective for protecting from mosquito bites [38]. Regarding the major compounds, the bibliographic data showed that limonene exhibits a good repellent activity [39]. This fact may explain the good repellant activity of PPI essential oil in our bioassays. Nevertheless, substances such as pinenes, limonenes, etc. may occur in EOs in one or more enantiomeric forms [17,40]. Since the enantioselectivity may play an important role in the activity of EOs, further phytochemical analysis and biological investigation of the individual components is needed in order to safely correlate the chemical composition with the repellent activity.

### 2.2. Isolation of Essential Oils’ Volatile Compounds by Centrifugal Partition Chromatography (CPC)

The next step of the study includes the fractionation of the essential oils obtained from the fruits of *J. oxycedrus* subsp. *deltoides* (JOX) and the needles of the *P. pinea* (PPI) by using Centrifugation Partition Chromatography. The goal here was both to develop an efficient method for the chromatographic analysis of EOs and to receive pure volatile compounds for further biological investigation regarding their repellent activity against *Ae. albopictus*. Based on the GC-MS analysis of the two selected EOs, the CPC analysis was focused on the recovery of enriched fractions of their main components i.e., *α*-pinene, *β*-myrcene and germacrene D from JOX and *α*-pinene, limonene, *β*-phelandrene, *β*-caryophyllene, and guaiol from PPI.

#### 2.2.1. Solvent System Selection and CPC Fractionation of Essential Oils

The first and most important step for a successful CPC separation is the selection of the appropriate biphasic solvent system. The bibliographic research on the analysis of essential oils by countercurrent chromatography revealed the presence of a limited number of specific scientific papers. The majority of the essential oils were fractionated using non-aqueous biphasic systems, which is justified by the strong hydrophobicity of their volatile constituents. Following the same approach, we created and tested several non-aqueous biphasic systems consisting of the solvents petroleum ether, heptane, acetonitrile, acetone and butanol in various combinations and proportions. The initial TLC tests of these systems revealed that the biphasic system Heptane/Acetonitrile/Butanol in the solvent ratio of 1.6/1.6/0.2 *v*/*v*/*v* showed the best distribution of the main components for both essential oils. This initial result was verified by GC-MS analysis (Figure S2a,b: Supplementary Materials) ware the distribution coefficients (Kd) and separation factors (a) of the target compounds have been calculated (Table 3).

In order to have an efficient separation using the CPC technique, K_D_ values between 0.2–5 and separation factors over 1.5 are needed [41]. Taking into account the distribution coefficient values (Table 3) we conclude that during the CPC analysis of JOX EO, *β*-myrcene will be eluted first, following by *α*-pinene and the last, germacrnene D. Regarding the separation quality, the measured separation factors showed that only *β*-myrcene will be completely separated (a > 1.5). Concerning *α*-pinene and germacrnene D (a = 1.46 < 1.5) there will be a partial overlap of the elution zones, thus giving fractions with pure *α*-pinene, fractions with pure germacrene D, and between those, some fractions with both compounds. As for the analysis of PPI EO, the compounds will be eluted in the following order: guaiol, *β*-phelandrene, limonene, *β*-pinene, *α*-pinene, *β*-caryophyllene. The calculated separation factors showed that guaiol will be completely separated from the other target compounds, limonene and *β*-phelandrene will be separated from the other compounds but only partially separated between them, while *α*-pinene will be eluted at almost the same time with *β*-pinene and *β*-caryophyllene (a < 1.5). Overall, the calculated K_D_ values and separation factors showed that the chosen biphasic system is capable to lead to an effective fractionation of the major compounds of both essential oils.

#### 2.2.2. Fractionation of *J. oxisedrus* subsp. *deltoides* Essential Oil

The CPC experiment was performed on an analytical column of 50 mL (FCPC50^®^) using the elution extrusion method in descending mode. The analysis started by establishing the hydrodynamic equilibrium of the two phases into the column (Sf = 58%). After sample injection (500 mg for each EO) the separation of the volatile compounds was achieved by initially passing 175 mL of the lower phase as a mobile phase (elution step), and then the column content was extruded using, as a mobile phase, the upper phase of the biphasic system in descending mode (extrusion step). The flow rate and rotation speed were set at 5 mL/min and 800 rpm, respectively, during the entire procedure, while the fraction collector was set to collect fractions every 1 min, resulting in a total of 45 fractions (of 5 mL) (see Section 3.5.). All obtained fractions were subjected to TLC analysis showing a successful fractionation of the EO (Figure S3: supplementary material). Subsequently, chosen CPC fractions were subjected to GC-MS analysis in order to identify the isolated compounds, as well as to assess their purity.

**Figure 1:**
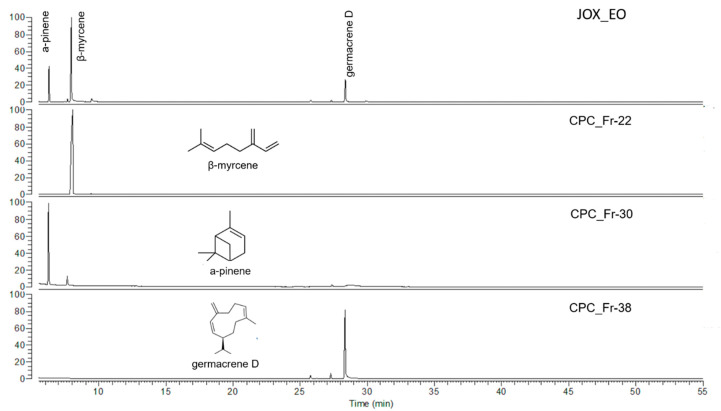
GC-MS analysis of essential oil (EO) from the fruits of *J. oxisedrus* subsp. *deltoides* and selected Centrifugal Partition Chromatography (CPC) fractions.

The GC-MS analysis of CPC fractions verified the elution order of the target compounds witch was calculated based on the K_D_ values. *β*-Myrcene was eluted first, and collected on fractions 17–26, *α*-pinene on fractions 26–36, while germacrene D was eluted during the extrusion step and collected in fractions 34–41. Regarding the purity of the isolated compounds, *β*-myrcene was recovered with high purity (>95%), germacrene D wiith a purity of approximately 85% (on fractions 37–40) while *α*-pinene was collected in mixture with its isomer *β*-pinene (Figure 1) in the fractions 26–30, almost in a pure form in the next three fractions (31–33) and in a mixture with germacrene D in the last fractions of its elution (34–36).

#### 2.2.3. Fractionation of *P. pinea* Essential Oil

The CPC analysis of the PPI essential oil was also performed in the analytical column of 50 mL by using the same biphasic system and following the same procedure as in the fractionation of JOX EO (see Section 3.5.). All collected fractions were initially analyzed by TLC (Figure S3: Supplementary Materials) and then their chemical compositions were determined by GC-MS.

**Figure 2:**
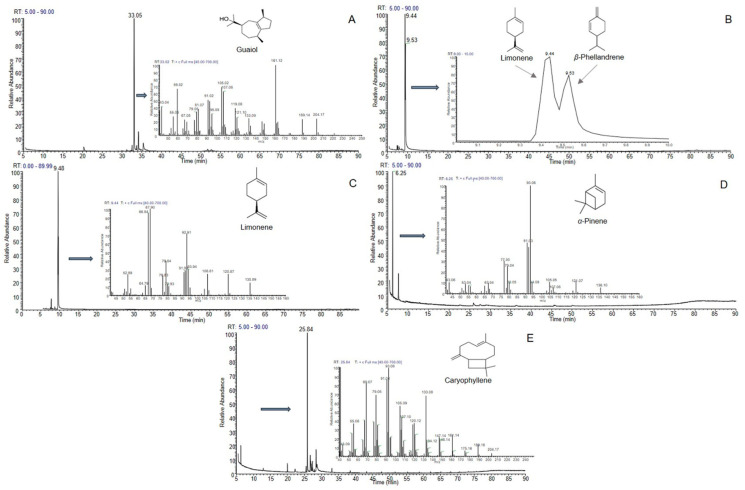
GC-MS analysis of CPC fractions obtained from the treatment of *P. pinea* (PPI) essential oil. (**A**): fr. 12–15; (**B**): fr. 23–27; (**C**): fr. 28–32; (**D**): fr. 34–35; (**E**): fr. 39–43.

Guaiol was the first of the target compounds that was eluted and collected in fractions 12–15. Based on GC-MS analysis, the purity of the isolated guaiol was found to be more than 80% (Figure 2A). *α*-Phellandren began to elute in fraction 23 together with limonene and continued eluting until fraction 27 (Figure 2B). The next five fractions (28–32) contained only limonene in a purity of more than 90% (Figure 2C). In order to clarify if the recovered limonene is (−) or (+), the isolated compound was subjected to optical rotation measurement. The optical rotation value was found to be ${\left[\alpha \right]}_{20}^{D}$ = −96 showing that the sample turns the polarized light counterclockwise, which is in accordance with the literature data available for (−)-limonene. Fractions 33–38 contained *α*-pinene as the major compound, together with its isomer, *β*-pinene. The ratios of the two compounds varied between the fractions starting from 70/30 in fraction 33–90/10 in fraction 36. This phenomenon was also observed during the fractionation of JOX EO and is justified from the similar distributions of these isomers in the two phases of the biphasic system. The last compound obtained from this analysis was *β*-caryophyllene, which was eluded during the extrusion step and recovered on fractions 39–43. The purity of this sesquiterpene was found to be approximately 70% (Figure 2E).

It is important to note that the CPC analysis of both EOs of *J. oxisedrus* subsp. *deltoides* fruits and *P. pinea* needles are presented here for the first time. The analysis of CPC fractions presents a successful fractionation of both EOs in a short time (Figure S3: supplementary material) leading to the recovery of the major compounds in pure form or in enriched fractions. Taking into account that countercurrent chromatography offers the advantage of ideal scaling-up from analytical to preparative and pilot processes [22] we conclude that this technique can be a good tool for the treatment of these demanding mixtures and the efficient recovery of high-value volatile compounds.

### 2.3. Repellent Activity of Volatile Compounds

The four isolated compounds, namely (−)-limonene, guaiol, *β*-myrcene and germacrene D and four simple mixtures of two compounds, namely *α*-pinene/*β*-pinene (ratio 70/30), *α*-pinene/germacrene D (ratio 65/45), (−)-limonene/*β*-pheladrene (ratios 55/45 and 70/30) obtained from the CPC treatment of PPI and JOX essential oils were evaluated for their repellent activity against *Ae. albopictus*. Table 4 presents the results over the mean number of landings on the uncovered area of the glove for 5 min and % repellency of tasted samples compared to the mean number of the landings of blank.

From the isolated compounds, (−)-limonene, guaiol and germacrene D showed “DEET-like” repellent activity. Guaiol and (−)-limonene, isolated from PPI EO presented the highest activity with 100% and 99.6% repellency respectively. Both compounds were isolated and tested for the first time against *Ae. albopictus*. On the other hand, the two tasted mixtures containing (−)-limonene and *β*-pheladrene at ratios of 55/45 and 70/30 also showed high repellent activity (98.1% and 99.1% repellency respectively). This fact led us to conclude that not only (−)-limonene, but also *β*-pheladrene is an active compound against the Asian tiger mosquito. Taking into account that *α*- and *β*-pinenes presented moderate activity (57.5% repellency of their mixture: Table 4) we conclude that mainly (−)-limonene (as the major compound) and, secondly, *β*-pheladrene and guaiol are responsible for the high repellent activity of the PPI essential oil (Table 2)

Germacrene D, isolated from JOX EO, presented high repellent activity (97.4% repellency: Table 4). In contrast, the isolated *β*-myrcene presented the lowest activity (31.4% repellency: Table 4) among all the tested samples. The low activity of *β*-myrcene (main compound of JOX EO) compared to the moderate activity of *α*-pinene explains the low repellent activity presented from the JOX essential oil. However, the CPC analysis of this EO resulted in the recovery of the active volatile compound germacrene D, which was isolated and tested for the first time against this mosquito species.

## 3. Materials and Methods

### 3.1. Chemicals

All solvents used for CPC, TLC and GC-MS analysis were of analytical grade. n-Heptane (n-Hept), Acetonitrile (CH_3_CN), Butanol (BuOH), were purchased from Carlo Erba Reactifs SDS (Val de Reuil, France) while c-Hexane (c-Hex) and CH_2_CL_2_ (DCM) from Fisher Scientific UK (Leicestershire, UK). Sulphuric Acid (H_2_SO_4_) and Vanillin standard (98%) were purchased from Sigma-Aldrich (Steinheim, Germany).

### 3.2. Plant Material

Wood parts of *P. nigra* J.F. Arnold subsp. *nigra* (Syn.: *P. pallasiana* Lamb.; *P. nigra* subsp. *pallasiana* (Lamb.) Holmboe; *P. pindica* Formánek) were collected from Mt. Smolikas, NW Greece (40°02′18′′N 20°53′19′′E), (Voucher specimen: SM 002). Wood parts of *P. heldreichii* Christ (Syn.: *P. leucodermis* Antoine; *P. heldreichii* subsp. *leucodermis* (Antoine) E. Murray) were collected from Mt. Smolikas, NW Greece (39°59′04′′N 20°50′41′′E), (Voucher specimen: SM 020). Needles of *P. pinea* L. were collected from the Botanic garden of Athens (Greece). (Voucher specimen: BGA 001). Fruits of *J. turbinata* Guss. (Syn.: *J. phoenicea* subsp. *turbinata* (Guss.) Nyman) were collected from Mt. Parnonas, Peloponnese, S Greece (37°20′06′′ N 22°48′25′′ E), (Voucher specimen: PARN 016). Fruits of *J. oxycedrus* subsp. *deltoides* (R.P. Adams) N.G. Passal. (Syn.: *J. deltoides* R.P. Adams) were collected from Mt. Parnonas, Peloponnese, S Greece (37°22′58′′ N 22°29′49′′ E), (the voucher specimen: PARN 015). The plant material was identified by the botanist Dr. E. Kalpoutzakis (NKUA, Athens, Greece) while the species names are according to Dimopoulos et. al. (2013, 2016) [42]. Specimens were stored in the herbarium of the Division of Pharmacognosy and Natural Products Chemistry, Department of Pharmacy, NKUA, Greece.

### 3.3. Isolation of the Essential Oils

The essential oils were prepared using a microwave-assisted hydrodistilation (MAHD). The instrument used for this purpose was a Start E Microwave Extraction System (Sorisole, Bergamo, Italy). A certain quantity of each plant (Table S1: Supplementary Materials) was placed in a 1 L round-bottomed distillation flask with 350 mL of water and was placed within the microwave oven cavity. A Clevenger apparatus was set on top, in order to collect the extracted essential oil. The protocol of the procedure was as follow: for the first two minutes we set the power at 1000 W, then for the next ten minutes the power was set at 700 W and for the last 15 min the power was set at 400 W. Overall, the procedure of the extraction lasted for 37 min. The oil was collected and dehydrated over anhydrous sodium sulfate to remove excess water and then weighted and stored in a vial at the freezer for further analysis.

### 3.4. GC-MS Analysis of the Essential Oils and CPC Fractions

Analyses of the essential oils and CPC fractions were performed on a Hewlett Packard 5973–6890 apparatus coupled to an HP 5973 mass spectrometer. The volatile compounds were separated in an HP-5MS capillary column (30 m × 0.25 mm; film thickness of 0.25 μm Agilent Palo Alto, CA, USA.) using Helium as carrier gas at a flow rate of 1 mL/min. The gas chromatograph oven temperature started at 60 °C and increased by 3 °C per minute until it reached 300 °C, where it remained for 10 min. The injected volume was 1 μL and the total analysis time for each sample lasted 90 min. The ion production method was an electron bombardment (EI-70 eV) while the identification of the compounds was based on a comparison of the obtained MS spectra with the mass spectra of the Wiley and NIST 2011 libraries and taking into account the elution order of the substances.

### 3.5. CPC Fractionation

Essential oils of *P. pinea* and *J. oxycedrus* subsp. *deltoides* were fractionated by CPC using for both experiments the biphasic solvent system composed of n-Heptane/Acetonitrile/Butanol 1.6/1.6/0.2 *v/v/v*. The appropriate biphasic system was selected according to the partition coefficient K_D_ of the target compounds. The K_D_ values were determined by GC-MS analysis using the following procedure: 10 mg of essential oil was added to the mixture of 3.4 mL of the biphasic system (1.6 mL n-Heptane, 1.6 mL Acetonitrile and 0.2 mL Butanol) and the solution was then mixed thoroughly by shaking. After the system equilibration, the two phases were separated and analyzed by GC-MS (Figure S2a,b: Supplementary Material). The K_D_ value was expressed as the peak area of the target compounds in the stationary phase divided by the one in the mobile phase (Table 3).

The experiments were performed on an FCPC Kromaton apparatus equipped with an analytical CPC column of 50 mL capacity (FCPC50^®^, Rousselet Robatel Kromaton, Annonay, France). For both analyses, the same protocol was followed. Initially, the column was filled with the upper stationary phase on descending mode at a flow rate of 8 mL/min and setting the rotation speed at 200 rpm. Then the rotation speed was increased to 800 rpm and the lower phase of the same system (mobile phase) was pumped at 5 mL/min on descending mode to equilibrate the two phases into the column (Sf was calculated at 0.58%). 500 μL of the essential oil were diluted in a mixture of the two phases (ratio 1:1 upper phase/lower phase) and injected via a 3-mL injection loop. Overall, a total of 35 fractions of 5 mL were collected using the lower phase as the mobile phase, and then the extrusion step was followed using the upper stationary phase as mobile phase resulting in the collection of 10 additional fractions of 5 mL. The rotation speed and flow rate were kept stable at 800 rpm and 5 mL/min, respectively, in descending mode, during the whole experiment.

### 3.6. TLC Analysis and Optical Rotation Measurement

All fractions were checked by Thin Layer Chromatography (TLC) on Merck 60 F254 (Billerica, MA, USA) pre-coated silica gel plates (20 × 10 cm). The compounds were eluted by c-Hex (100% *v*/*v*) system. TLC plates were sprayed with a solution of equal volumes of 5% vanillin in methanol and 5% *v/v* sulfuric acid in methanol and finally revealed by heating.

The received limonene was analyzed on a polarimeter PERKIN ELMER 341 (Hopkinton, MA, USA) in order to measure its optical rotation. The sample diluted in DCM at a concentration of 0.3 g/100 mL revealed an observed rotation of −0.29. The specific rotation ${\left[\alpha \right]}_{20}^{D}$ was calculated according to the formula: ${\left[\alpha \right]}_{20}^{D}$ = β * 100/μ * d (were β: the observed rotation, μ: cell length in dm and d: sample concentration in g/100 mL) at 20 °C and was found to be −96.

### 3.7. Mosquitoes and Repellence Bioassays

Adult *Ae. albopictus* mosquitoes were obtained from the laboratory colony of the Benaki Phytopathological Institute (Kifissia, Greece) [43]. The laboratory colony is maintained at 25 ± 2 °C, 80% relative humidity, and 16/8-h light/dark photoperiod [43].

For the in vivo determination of the repellent activity of the selected EOs and isolated compounds and mixtures, the evaluation was based on the number of mosquito landings on human skin at a dose equivalent to 0.2 μL/cm^2^ diluted with dichloromethane (DCM) [17]. Control experiments with compound-free DCM solvent or DEET treatments (negative and positive controls, respectively) were also conducted. Each treatment was repeated 5–9 times on two human volunteers. Landing numbers were converted to repellence indices using the equation RI = [1 − T/C] × 100, where C was the number of landings in control and T the number of landings in treatment.

## 4. Conclusions

In the current study an effective CPC-based protocol for the recovery of volatile compounds with repellent activity against *Ae. Albopictus* was developed. The initial biological screening of five EOs of *Pinus* and *Juniperus* species revealed that the essential oil obtained from needles of *P. pinea* presented significant ability to repel the Asian tiger mosquito. The EOs obtained from the wood part of *P. nigra* subsp. *nigra* and *P. hedreichii* and the fruit of *J. oxycedrus* subsp. *deltoides* are tested herein for the first time and present moderate repellent activity. A rapid and effective CPC method for the isolation of the main terpenes of the EOs of *P. pinea* and *J. oxycedrus* subsp. *deltoides* was developed. The CPC analyses of both EOs are presented here for the first time and resulted in the successful isolation of (−)-limonene, guaiol, *β*-myrcene and germacrene D as well in the recovery of simple mixtures of (−)-limonene/*β*-phellandrene, *α*-pinene/*β*-pinene and *α*-pinene/germacrene D. From them, (−)-limonene, guaiol, and germacrene D presented high repellent activity against *Ae. albopictus* while *β*-myrcene showed only weak activity.

## Figures and Tables

**Table 1:** Major constituents of essential oils (EOs) identified by gas chromatography–mass spectrometry (GC-MS). (PNI: *P. nigra* subsp. *nigra*, PHE: *P.heldreichii*, PPI: *P. pinea*, JTU: *J. turbinata* fruits, JOX: *J. oxycedrus* subsp. *Deltoides*, RI: retention index).

Constituents	RI	PNI	PHE	PPI	JTU	JOX
*α*-Pinene	930	32.82	1.49	9.50	81.72	15.65
*β*-Pinene	970	0.31	ND	3.77	2.77	1.05
*β*-Myrcene	981	0.07	ND	2.46	3.65	43.53
Limonene	1021	1.70	3.48	45.05	1.70	4.00
*β*-Phelladrene	1024	ND	ND	12.79	0.16	ND
cis-*β*-Terpineol	1136	ND	47.41	ND	ND	ND
trans-*β*-Terpineol	1151	ND	6.56	ND	ND	ND
*α*-Terpineol	1177	ND	9.19	ND	0.58	ND
Longifolene	1399	ND	11.88	1.12	ND	ND
*β*-Caryophyllene	1410	0.28	ND	6.40	0.99	1.76
*γ*-Muurolene	1468	4.93	ND	ND	ND	0.32
Germacrene D	1475	0.61	ND	3.29	ND	23.77
*δ*-Cadinene	1514	5.23	ND	0.73	ND	2.74
Guaiol	1593	ND	ND	4.25	ND	ND
Cembrene	1929	ND	6.26	ND	ND	ND
Thunbergol	2057	ND	9.61	ND	ND	ND
Sandaracopimarinal	2172	18.10	ND	ND	ND	ND
Other minor compounds *		28.06	2.52	8.52	6.62	5.86
Total identified (%)		92.11	98.40	97.88	98.19	98.68

* The percentage of the other minor compounds identified in the essential oils. All identified compounds are presented in Table S1 (supplementary material); ND = not detected.

**Table 2:** Repellent activity of essential oils against *Ae. albopictus* adults in human hand landing assays. (JOX: *J. oxycedrus* subsp. *deltoids*; JTU: *J.* *turbinate*; PHE: *P. heldreichii*; PPI: *P. pinea*; PNI: *P. nigra* subsp. *nigra*).

EOs	Mean Number of Landing ± SE	% Mean Repellency ± SE
JOX	38.6 ± 4.4	41.8 ± 6.6
JTU	35.7 ± 3.6	42.0 ± 4.5
PHE	42.5 ± 7.2	36.1 ± 10.8
PPI	11.7 ± 2.4	82.4 ± 3.6
PNI	51.7 ± 2.9	22.3 ± 4.4
DEET	0	100
Control	66.5 ± 2.9	-

**Table 3:** Partition coefficients (K_D_) values and separation factor (a) of the main components of JOX and PPI EOs using the biphasic system heptane/acetonitrile/butanol 1.6/1.6/0.2 *v*/*v*/*v*.

EO	Rt	Compound	K_D_	a
**JOX**	6.24	*α*-Pinene	1.46	1.45/1.81
	7.91	*β*-Myrcene	0.81	1.81
	28.47	Germacrene D	2.12	1.45
**PPI**	6.22	*α*-Pinene	1.39	1.17/1.2
	7.62	*β*-Pinene	1.19	1.75/1.17
	9.40	Limonene	0.68	1.75/1.4
	9.49	*β*-Phelandrene	0.47	1.4/4.2
	25.82	Cariophylene	1.76	1.27
	33.06	Guaiol	0.11	4.2

**Table 4:** Repellent activity of pure compounds and mixture of two compounds (obtained by the Centrifugal Partition Chromatography (CPC) analysis of JOX and PPI essential oils) against *Ae. albopictus* adults in human hand landing assays.

Treatment		Mean Number of Landing ± SE	% Mean repellency ± SE
Isolated compounds	Guaiol	0	100
	*β*-Myrcene	47.3 ± 2.6	32.7 ± 3.3
	Germacrene D	1.7 ± 0.7	97.5 ± 1.1
	(−)-Limonene	0.3 ± 0.2	99.5 ± 0.3
Mixtures of two compounds	*α*-Pinene/*β*-Pinene 70/30	30.3 ± 5.5	56.9 ± 7.9
	*α*-Pinene/Germacrene D 65/45	7.3 ± 1.2	89.6 ±1.7
	(−)-Limonene/*β*-Pheladrene 55/45	1.3 ± 0.7	98.1 ± 0.9
	(−)-Limonene/*β*-Phelladrene 70/30	0.7 ± 0.7	99.1 ± 0.9
Pos. control	N,N-diethyl-3-methylbenzamide (DEET)	0	100
Neg control	Dichloromethane (DCM)	70.4 ± 2.1	-

## Data Availability

Not applicable.

## Supplementary Materials

The following are available online at, Figure S1: TIC Chromatogram of PNI (a), PHE (b), PPI (c), JTU (d) and JOX (e), Figure S2: GC-MS chromatograms of upper (A) and lower phases (B) of JOX (a) and PPI (b) treated with the biphasic system Hept./ACN/BuOH 1.6/1.6/0.2 (*v/v/v*), Figure S3: TLC chromatograms of CPC fractions from analyses of JOX and PPI essential oils, Table S1: Species, plant parts, weight of extraction material, volume and yield of recovered EOs, Table S2: Identified compounds of PNI, PHE, PPI, JTU and JOX essential oils.

## Sample Availability

Essential oils and CPC fractions are available from the authors.
