# Supplementary material for: Isolation of Volatile Compounds with Repellent Properties against Aedes albopictus (Diptera: Culicidae) Using CPC Technology
Source: Molecules. 2021 May 21;26(11):3072. doi: 10.3390/molecules26113072 (PMC8196645; doi:10.3390/molecules26113072)
Supplement: Supplementary file 1 [file molecules-26-03072-s001.zip › molecules-1176729-supplementary.pdf]

Isolation of Volatile Compounds with Repellent Properties against *Aedes albopictus* (Diptera: Culicidae) using CPC technology

Anastasia Liakakou[1#], Apostolis Angelis[1#], Dimitrios P. Papachristos[2], Nikolas Fokialakis[1], Antonios Michaelakis[2], Leandros A. Skaltsounis[1*]

[1]Laboratory of Pharmacognosy and Natural Products Chemistry, School of Pharmacy, University of Athens, Panepistimioupoli, Zografou, 15771 Athens

[2]Benaki Phytopathological Institute, Scientific Directorate of Entomology and Agricultural Zoology, 14561, Kifissia;

[#]Equal contribution

*Corresponding authors: Leandros A. Skaltsounis , Email; skaltsounis@pharm.uoa.gr

Contents

Table S1. Species, plant parts, weight of extraction material, volume and yield of recovered EOs.

Table S2: Identified compounds of PNI, PHE, PPI, JTU and JOX essential oils

Figure S1a: TIC Chromatogram of PNI

Figure S1b: TIC Chromatogram of PHE

Figure S1c: TIC Chromatogram PPI

Figure S1d: TIC Chromatogram of JTU

Figure S1e: TIC Chromatogram of JOX

Figure S2a: GC-MS chromatograms of upper (A) and lower phases (B) of JOX treated with the biphasic system Hept./ACN/ BuOH 1.6 / 1.6 / 0.2 (v/v/v)

Figure S2b: GC-MS chromatograms of upper (A) and lower phases (B) of PPI treated with the biphasic system Hept./ACN/ BuOH 1.6 / 1.6 / 0.2 (v/v/v)

Figure S3: TLC chromatograms of CPC fractions from analyses of JOX and PPI essential oils

Table S1. Information regarding the species, plant parts, weight of extraction material, volume and yield (%v/w of tested materials) of recovered EOs.

Species	Plant Part	Weight of row material (gr)	Volume of EO (ml)	% v/w
P. nigra subsp. *nigra*	wood	141	0.2	0.07
P. heldreichii	wood	88	0.6	0.57
P. pinea	needles	14603	4	0.03
J. turbinata	fruits	330	6.5	1.96
J. oxycedrus subsp. *deltoides*	fruits	251	6.7	2.66

Table S2: Constituents listed in order of elution from a HP-5 MS column. (PNI: *P.nigra* subsp. *nigra*, PHE: *P.heldreichii*, PPI: *P. pinea*, JTU: *J.turbinata*, JOX: *J.oxycedrus* subsp. *deltoides*).

aConstituents	KI	PNI	PHE	PPI	JTU	JOX
Tricyclene	921	-	-	-	0.48	-
α-Pinene	930	32.82	1.49	9.50	81.72	15.65
Camphene	944	0.71	-	-	0.85	0.12
Sabinene	966	0.12	-	0.59	0.52	0.21
β-Pinene	970	0.31	-	3.77	2.77	1.05
Myrcene	981	0.07	-	2.46	3.65	43.53
α-Phelladrene	993	-	-	0.56	-	-
o-Cymene	1019	0.38	-	-	-	-
Limonene	1021	1.70	3.48	45.05	1.70	4.00
β-Phelladrene	1024	-	-	12.79	0.16	-
δ-3-Carene	1027	-	-	-	0.21	-
(E) -β – Ocimene	1041	-	-	1.45	-	-
Terpinolene	1079	-	-	0.70	-	0.87
Nonanal	1092	0.50	-	-	-	-
α-Fenchol	1108	1.02	-	-	-	-
α-Campholenal	1120	2.26	-	-	0.37	-
cis-β-Terpineol	1136	-	47.41	-	-	-
Camphen hydrate	1140	0.41	-	-	-	-
trans-β-Terpineol	1151	-	6.56			
Pinocarvone	1152	1.12	-	-	-	-
p-Mentha1,5diene-8ol	1162	0.89	-	-	-	-
Terpinene4ol	1165	0.86	-	-	0.21	0.23
α-Terpineol	1177	-	9.19	-	0.58	-
Verbenone	1199	0.08	-	-	0.55	-
Carveol trans	1211	-	-	-	-	-

Carvone	1237	0.18	-	-	-	-
Bornyl acetate	1276	-	-	-	-	0.24
Azulene	1289	0.18	-	-	-	-
δ-Elemene	1327	-	-	0.51	-	-
α-Terpinyl acetate	1339	-	-	-	0.27	-
α-Cubebene	1340	0.07	-	-	-	0.22
Citronellyl acetate	1342	-	-	-	-	0.20
α-Longipinene	1343	-	0.77	-	-	-
Longicyclene	1361	-	0.18	-	-	-
α-Ƴlangene	1362	0.53	-	-	-	-
α-Copaene	1364	1.06	-	-	-	0.43
Geranyl acetate	1370	-	-	-	-	0.46
β-Bourbonene	1378	1.14	-	-	-	
β-Elemene	1379	-	-	-	0.15	0.12
Longifolene	1399	-	11.88	1.12	-	-
β-Caryophyllene	1410	0.28	-	6.40	0.99	1.76
β-Copaene	1423	0.59	-	-	-	0.10
α-Guaiene	1430	-	-	0.30	-	
Z-*β*-Farnesene	1434	-	-	-	-	0.36
α-Humulene	1442	-	-	1.12	0.53	1.58
γ-Muurolene	1468	4.93	-	-	-	0.32
α-Amorphene	1474	0.35	-	-	-	
Germacrene D	1475	0.61	-	3.29	-	23.77
β-Selinene	1487	-	0.77	-	-	-
δ-Selinene	1490	-	-	3.29	-	-
trans-Muurola 4 (14) 5 diene	1492	-	-	-	2.40	-
γ-Amorphene	1489	1.06	-	-	-	-
α-Selinene	1490	-	0.21	-	-	-
α-Muurolene	1499	1.11	-	-	0.08	0.46
δ-Amorphene	1505	0.17	-	-	-	-
γ-Cadinene	1505	2.54	-	-	-	0.26
δ-Cadinene	1514	5.23	-	0.73	-	2.74
trans-Calamenene	1518	1.04	-	-	-	-
α-Cadinene	1530	0.31	-	-	-	-
α-Calacorene	1539	0.66	-	-	-	-
β-Calacorene	1557	0.28	-	-	-	-
Guaiol	1593	-	-	4.25	-	-
1-epi Cubenol	1612	0.56	-	-	-	-
epi-*α*-Murrolol	1632	0.68	-	-	-	-
α-Cadinol	1644	1.77	-	-	-	-
Cadalene	1669	0.81	-	-	-	-

Cembrene	1929	-	6.26	-	-	-
(3E)-Cembrene A	1943	-	0.59	-	-	-
Thunbergol	2057		9.61			
Sandaracopimarinal	2172	18.10	-	-	-	-
Sandaracopimarinol	2268	3.45	-	-	-	-
Abietal	2300	1.17	-	-	-	-
Total identified (%)		92.11	98.4	97.88	98.19	98.68
Total number of compounds		43	13	18	19	23

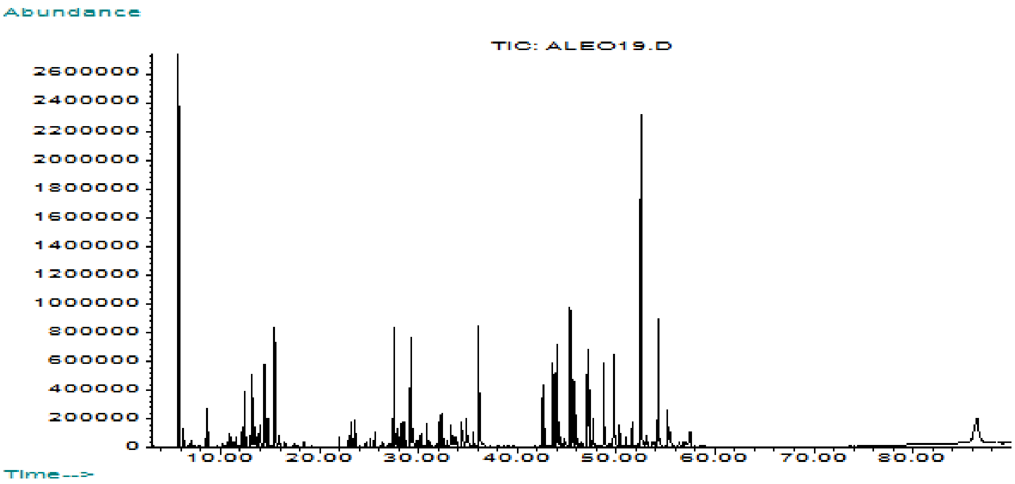

Figure S1a: TIC Cromatogram of *Pinus nigra* subsp. *nigra* (wood)

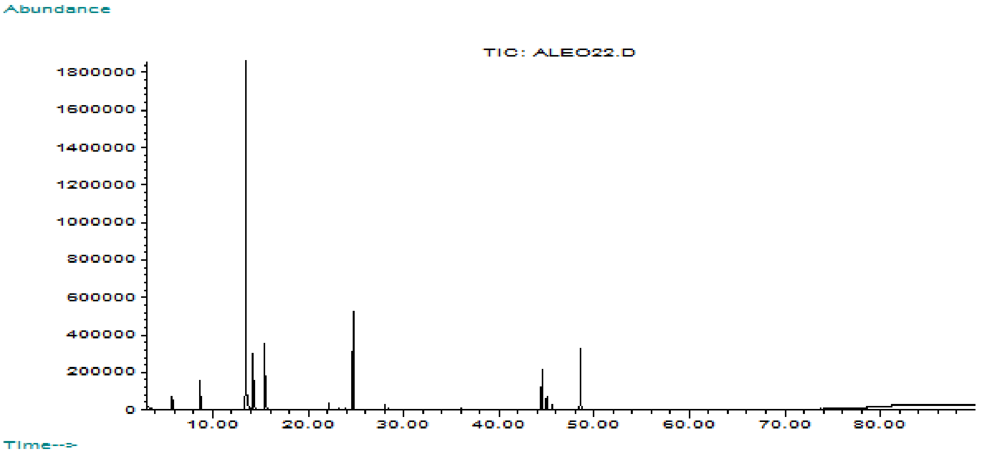

Figure S1b: TIC Chromatogram of *Pinus heldreichii* (wood)

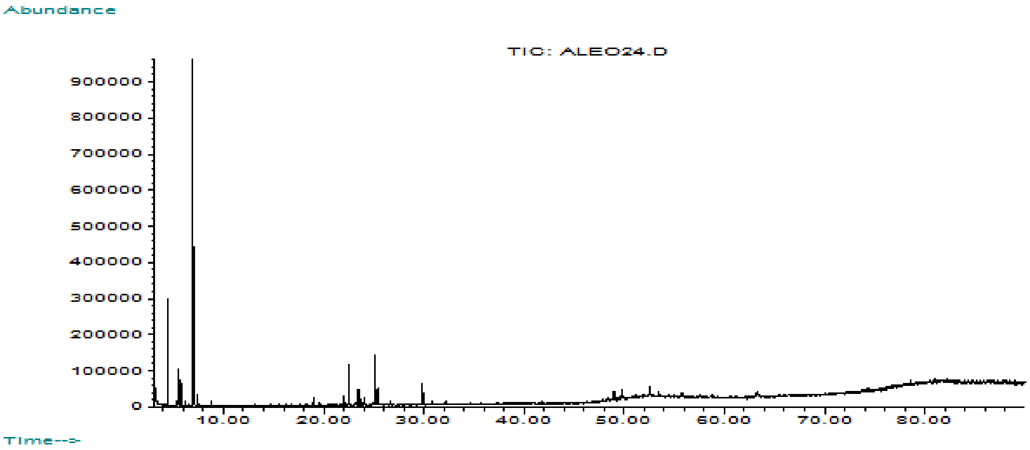

Figure S1c: TIC Chromatogram *Pinus pinea* (needles)

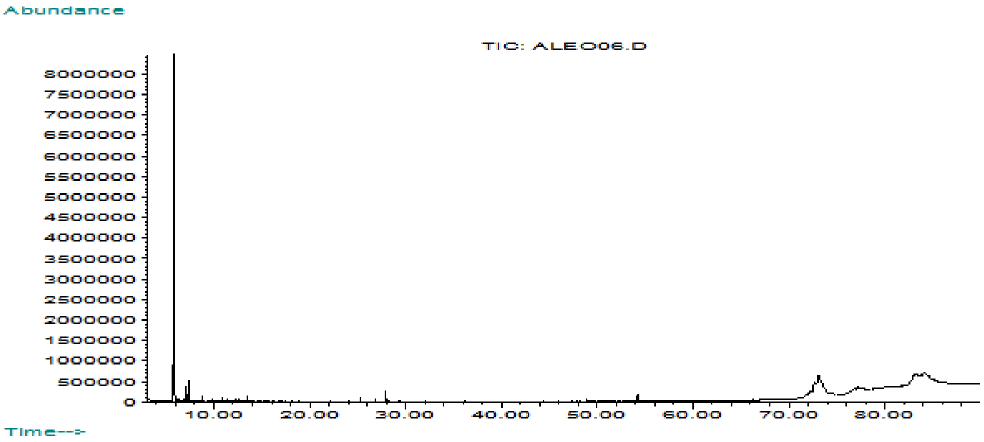

Figure S1d: TIC Chromatogram of *Juniperus turbinata* (fruits)

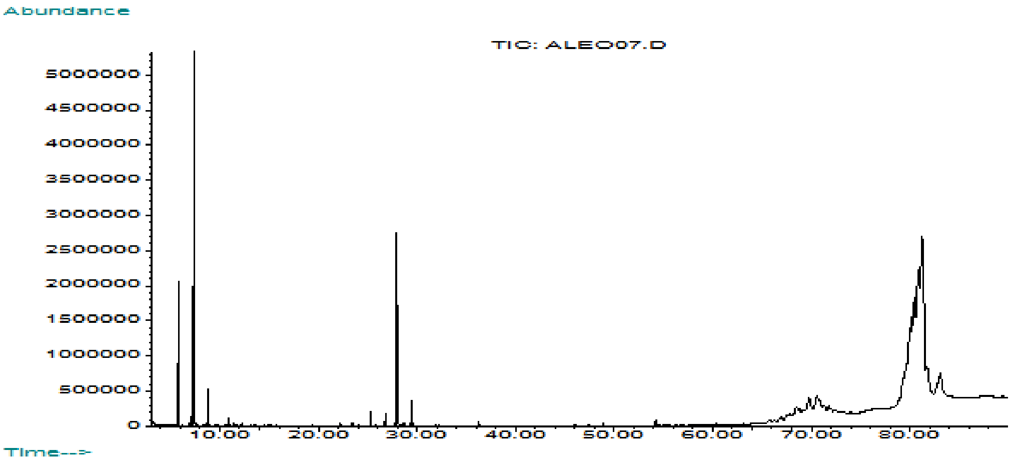

Figure S1e: TIC Chromatogram of *Juniperus oxycedrus* subsp. *deltoides* (fruits)

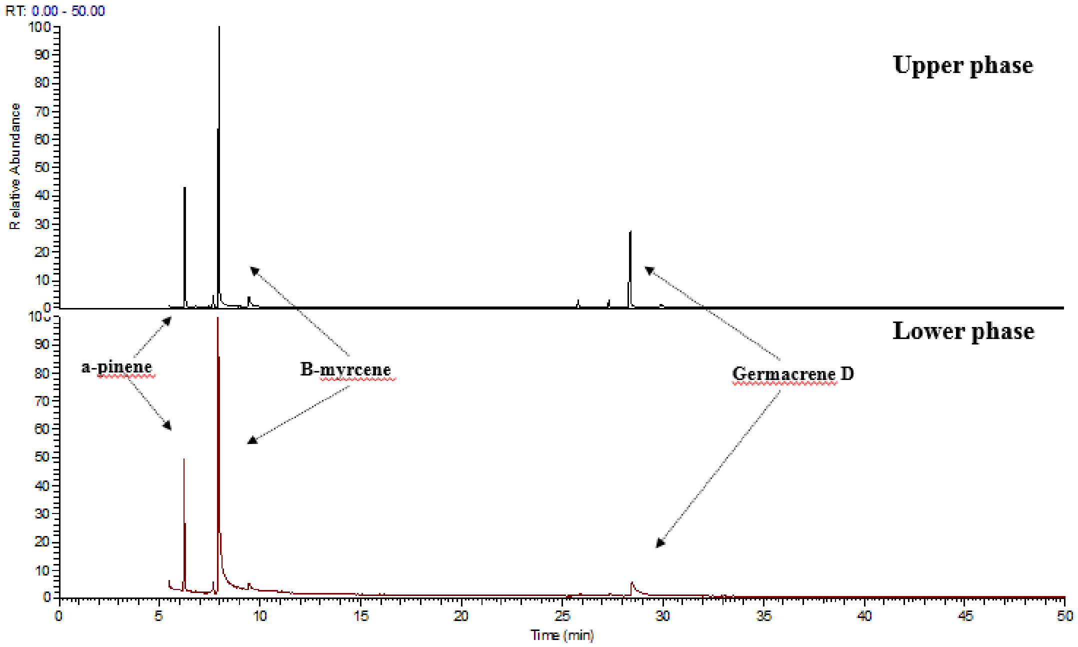

Figure S2a: Upper (A) and lower phase (B) GC-MS chromatograms obtained from *J.oxycedrus* subsp. *deltoides* essential oil with Heptane / Acetonitrile / Butanol biphasic system 1.6 / 1.6 / 0.2 v

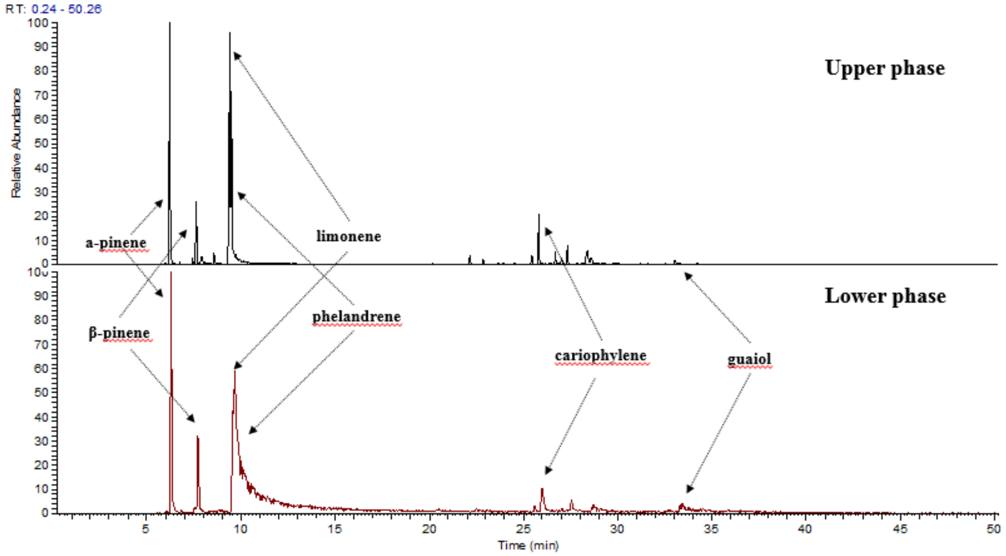

Figure S2b: Upper (A) and lower phase (B) GC-MS chromatograms obtained from *P. pinea* essential oil with Heptane / Acetonitrile / Butanol biphasic system 1.6 / 1.6 / 0.2 v

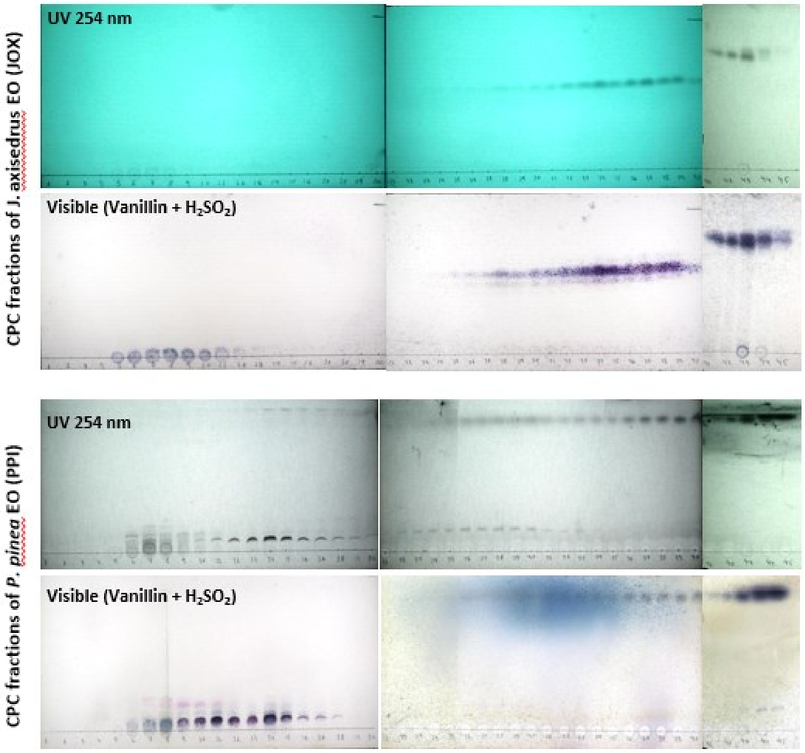

Figure S3: TLC chromatograms of fractions from CPC analyses of the essential oils of *J.oxycedrus* subsp. *deltoides* (upper) and *P. pinea* (lower) observation at UV-254 nm and visible after spraying with vanillin sulfate solution and heating
